# Sirt3 Protects Against Thoracic Aortic Dissection Formation by Reducing Reactive Oxygen Species, Vascular Inflammation, and Apoptosis of Smooth Muscle Cells

**DOI:** 10.3389/fcvm.2021.675647

**Published:** 2021-05-21

**Authors:** Lin Qiu, Shaolei Yi, Tingting Yu, Yan Hao

**Affiliations:** ^1^Shanghai Institute of Nutrition and Health, Chinese Academy of Sciences, Shanghai, China; ^2^Department of Cardiology, Shandong Provincial Hospital Affiliated to Shandong First Medical University, Jinan, China; ^3^Department of Dermatology, Huashan Hospital, Fudan University, Shanghai, China

**Keywords:** aortic dissection, sirt3, SMC, inflammation, apoptosis

## Abstract

Sirtuin3 (Sirt3) is a histone deacetylase involved in the regulation of many cellular processes. Sirt3 deficiency is known to increase oxidative stress. Reactive oxygen species (ROS) promote degradation of the extracellular matrix and vascular smooth muscle cell (VSMC) apoptosis. Reducing oxidative stress by Sirt3 overexpression could have therapeutic potential for limiting thoracic aortic dissection (TAD) development. We hypothesized that Sirt3 deficiency could increase the risk for TAD by decreasing ROS elimination and that Sirt3 overexpression (Sirt3^OE^) could provide an alternative option for TAD treatment. Mice with TAD had significantly lower Sirt3 expression than normal subjects. Sirt3 KO mice exhibit significantly increased TAD incidence rate and increased aortic diameters. Moreover, Sirt3 overexpression reduced Ang II-induced ROS production, NF-kB activation, and apoptosis in human aortic smooth muscle cells (HASMCs). Sirt3 overexpression attenuated aneurysm formation and decreased aortic expansion. In conclusion, our data showed that Sirt3 deficiency increases susceptibility to TAD formation by attenuating anti-ROS effects and increasing VSMC apoptosis and vascular inflammation.

## Introduction

Thoracic aortic dissection (TAD) is the most critical and catastrophic disease of all types of aortic syndrome, which has a rapid onset and a dangerous prognosis (1). When aortic intima has a rupture caused by various reasons, then blood flows into the aortic wall through the rupture, so that the middle layer is peeled from the adventitia to form two cavities. The blood fluid forms a dissecting hematoma along the false cavity and along the aorta (2). The wall is extended, and patients often die from pericardial tamponade or massive chest hemorrhage. The treatment of this fatal vascular disease requires timely interventional treatment and surgical repair. However, drug treatment has not yet achieved good clinical effects. The fundamental reason is that the underlying pathological mechanism leading to TAD is still unknown (3). Therefore, to better clarify the cellular and molecular mechanisms of the occurrence and development of TAD is essential to developing effective prevention and treatment methods.

Analysis of TAD revealed that the degeneration of the aortic media was an important pathological basis, including the apoptosis of smooth muscle cells, the fragmentation of elastic fibers, the degradation of collagen fibers, and the infiltration of inflammatory cells (3, 4). Increasing evidences have shown that oxidative stress plays a vital role in the pathological process of aortic media degeneration. Excessive oxidative stress turns the delicate balance between the generation and elimination of reactive oxygen species (ROS) into redundant ROS accumulation. Excessive ROS concentration leads to oxidative stress damage, including enhanced lipid peroxidation of biofilms and intracellular calcium ion overload, protein denaturation, reduced enzyme activity, DNA breakage, and chromosome aberration, and induces the production of inflammatory mediators, resulting in irreversible cell damage or death (5). In addition, ROS can also affect the expression of matrix metalloproteinases (MMP) and induce apoptosis of aortic smooth muscle cells (SMCs), which are the main pathological changes in diseases such as TAD and aneurysm. Angiotensin-II (Ang-II) perfusion is an important method for inducing mouse models of TAD. One of the mechanisms is that Ang-II can increase the production of ROS in vascular cells and inflammatory cells, thus aggravating the oxidative stress injury of vascular wall, and finally promoting the formation of aortic dissection or aneurysm (6).

Studies have shown that ursodeoxycholic acid prevented Ang II-induced NADPH subunit expression in an Nrf2-dependent manner, rescuing the activity of oxidoreductase, thereby inhibiting the apoptosis of VSMCs, and finally preventing the formation of TAD (3). In addition, BOP1 is an important structural element in the process of ribosome biogenesis, and its expression is significantly decreased in dissection patients and mice; further, after knocking out the BOP1 gene in HASMCs, it was found that the smooth muscle contractile protein α-SMA and MLC were downregulated, induce SMC apoptosis, and promote the production of ROS (7). Collectively, these data indicate that oxidative stress is closely associated with the occurrence and development of TAD.

Sirtuins are a class of nicotinamide adenine dinucleotide (NAD^+^)-dependent class III histone deacetylases. Sirtuins are involved in the regulation of many cellular processes, including circadian rhythm, metabolism, gene transcription, cell cycle, and apoptosis (8). Sirt3 has a wide range of substrates and is closely related to cardiovascular diseases. Sirt3 is mainly located in the mitochondrial matrix and participates in almost all physiological activities of mitochondria, including energy metabolism, production and detoxification of ROS, regulation of electron transport chains, maintenance of mitochondrial membrane potential, mitochondrial dynamics, and regulation of apoptosis (9). Isocitrate dehydrogenase 2 (IDH2) is located in the mitochondria and can catalyze the oxidative decarboxylation of isocitrate to produce ketoglutarate and NADPH. Sirt3 can deacetylate IDH2 and increase the level of NADPH, thereby enhancing the elimination of ROS (10). Studies have shown that the level of mitochondrial protein hyperacetylation in Sirt3 KO rats is significantly increased, and the mitochondrial metabolism rate is high, which is the key site that causes the increased aggregation of ROS (11). Under pathological conditions, Sirt3 deletion-mediated mitochondrial disorders are involved in the development of human cardiovascular diseases. Sirt3 can increase the transcription of MnSOD and catalase by deacetylating the transcription factor Fox03a, while Sirt3 KO transgenic mice show reduced MnSOD and catalase activities (12). Sirt3 regulates the level of oxidative phosphorylation by deacetylating the complexes of the mitochondrial respiratory chain, promotes ATP synthesis, and reduces ROS production. Together, Sirt3 can not only enhance the removal of ROS by increasing the activity of related antioxidant enzymes and protein expression but also reduce the production of ROS, which has an important defense effect on oxidative stress (13). In conclusion, oxidative stress is closely related to the pathogenesis of TAD, and many links of oxidative stress reaction are regulated by Sirt3. However, whether Sirt3 is involved in the occurrence and development of TAD has not been reported.

In this study, we observed that the expression of Sirt3 was significantly downregulated in the aortic tissues of BAPN and Ang II-induced TAD mice. The deficiency of the Sirt3 gene promoted the progression of TAD and increased the rupture rate of dissection vessels and the mortality of mice. Further mechanism studies have shown that Sirt3 loss participates in the occurrence and development of TAD by enhancing the inflammatory response of blood vessel walls, ROS production, and promotion of SMC apoptosis. However, overexpression of Sirt3 can reverse the abovementioned pathological changes. Therefore, we speculated that Sirt3 deficiency promotes the pathogenesis of TAD by inducing oxidative stress damage, and suggested that activating Sirt3 activity may be a potential strategy for TAD treatment.

## Materials and Methods

### Animals

Sirt3 KO mice were purchased from the Chinese Academy of Sciences. Four-week-old male C57BL/6J mice were purchased from the Animal Center of JSJ Company (Shanghai, China). The age of the mice was a key factor in BAPN modeling; only when the mice were in the early stage of rapid growth could BAPN promote the degradation of elastic fibers. Then, combined with Ang II treatment, aortic dissection was induced (14, 15). We only studied male mice in our experiments, because male mice could develop aortic dissection with high incidence and male mice had less sex hormone variations (16–18). SMC-enhanced adeno-associated virus (AAV) packaging was purchased from HANHEN (Shanghai, China). AAV-GFP-Sirt3 and AAV-GFP viruses (1010 plaque-forming units) were injected into the tail veins of C57BL/6J mice following the administration of β-aminopropionitrile monofumarate in the drinking water [β-aminopropionitrile monofumarate (BAPN), 1 g/kg per day, Sigma-Aldrich, St Louis] (19) and Angiotensin II treatment by micro pumps (Model 1004, Alzet, CA, USA) continuously at a dose of 1,000 ng/kg/min (20) for 4 weeks to induce aortic dissection (*n* = 16 per group). Simultaneously, mice were fed a normal diet for 4 weeks. Four weeks later, the mice were anesthetized with 1% pentobarbital sodium for surgery. Mice were kept under a 12-h light/dark cycle at 23°C with access to food (normal diet; LQ01; Qinglongshan) and water *ad libitum*. Cage bedding was from Qinglongshan Animal Center (Nanjing, China). All animal experiments were performed in specific pathogen-free barrier conditions in accordance with institutional guidelines and a protocol approved by the Committee on the Ethics of Animal Experiments.

### Cell Culture and Treatment

Human aortic smooth muscle cells (HASMCs) were purchased from ScienCell Research Laboratories (#6110) and maintained in DMEM (Gibco) supplemented with 10% (v/v) FBS (Gibco). Confluent cells (80–90%) were treated with Ang II (100 nM; Sigma-Aldrich).

### Transient Transfection Assay

Sirt3 siRNA was purchased from Santa Cruz Biotechnology (sc-61555). Sirt3 (Gene ID: 23410) cDNA was purchased from GENEWIZ, Inc. (Suzhou, China). Human Sirt3 cDNA was cloned into pcDNA3.1 by GENEWIZ. HASMCs were transfected with the indicated plasmids or siRNA using Lipofectamine 3000 reagent (Invitrogen) according to the manufacturer's recommendations. Cells were cultured for 24 h after transfection and before drug treatment.

### Measurement of ROS Formation

Superoxide production in cells was detected by 2′,7′-dichlorofluorescein diacetate (DCFH-DA) staining, which is oxidized by ROS to form the highly fluorescent 2′,7′-dichlorofluorescein (DCF). Briefly, HASMCs were treated as described above, after which the cells were washed twice with PBS and incubated with 5 μM of DCFH-DA for 30 min and washed twice with PBS. Fluorescence was measured with a NiKOn TE2000 Inverted Microscope and quantified using Image-Pro Plus analysis software. Fluorescence was measured through confocal microscope (Zeiss LSM 410) and quantified using Image-Pro Plus analysis software.

### Protein Extraction and Western Blot

Whole-cell lysates were obtained by resuspending cell pellets in RIPA buffer (50 mmol/L Tris pH 7.4, 150 mmol/L NaCl, 1% Triton X-100) with a freshly prepared protease inhibitor (Thermo Fisher, Waltham, MA). Equal amounts of cell lysates were loaded and separated on 15 or 10% SDS-poly-acrylamide gels and transferred onto polyvinylidene fluoride membranes. The membranes were blocked with 5% FBS and incubated with the following specific primary antibodies at 4°C overnight: anti-p65 (no. 8242, CST, Danvers, MA), anti-p-p65 (no. 3033, CST, Danvers, MA), anti-p38 (no. 8690, CST, Danvers, MA), anti-p-p38 (no. 4511, CST, Danvers, MA), anti-caspase3 (no. 9662, CST, Danvers, MA), anti-cleaved-caspase3 (no. 9661, CST, Danvers, MA), anti-Sirt3 (no. 2627, CST, Danvers, MA), and anti-GAPDH (no. AP0063, Bioworld, Nanjing, China). Then, the membranes were incubated with the corresponding secondary antibodies.

### TUNEL Staining

The TUNEL assay was performed with a one-step TUNEL kit (Beyotime Institute of Biotechnology, Haimen, China), according to the manufacturer's protocol. In brief, cells were fixed were fixed at room temperature in 4% (w/v) paraformaldehyde for 1 h. After specific labeling, the cells were exposed to darkness at room temperature with PBS for 5 min and repeated three times. Then, DAPI (5 mg/mL) was added on the surface of the cell. TUNEL-positive cells were defined as those with fluorescein-dUTP staining present. Then, 20 different fields were randomly selected under magnification (×40 oil) to count the number of apoptotic cells by confocal microscopy (FluoView 1000; Olympus Corporation).

### HE and EVG Staining

The HE and EVG staining was performed with staining kits (no. ab150667, ab245880, abcam), according to the manufacturer's protocol. In short, for EVG staining, the slide was placed in the elastic staining solution for 15 min and rinsed under running water until there are no excess stains on the slide. The slides were then soaked in the identification solution 15–20 times and rinsed under running water. The slide is then examined under a microscope to ensure proper identification. The slide was flushed with running water. The slide was then placed in sodium thiosulfate solution for 1 min. The slide was flushed with running water and finally dyed with Van Giesen solution for 2–5 min, rinsed twice with 95% alcohol, and dehydrated with anhydrous alcohol. For HE staining, an appropriate amount of hematoxylin was taken to completely cover the tissue sections and incubated for 5 min. The slides were washed twice with distilled water to remove excess dye. The tissue sections were covered completely with sufficient nuclear stain and incubated for 10–15 s. The slides were washed with distilled water twice and soaked in anhydrous alcohol to absorb any excess alcohol. The tissue sections were covered completely with an appropriate amount of eosin solution and incubated for 2–3 min. Rinse the slides with anhydrous alcohol. Dehydrate and seal the tablets.

### Statistical Analysis

All experiments were repeated at least six times. Results are presented as the mean ± SEM and analyzed in GraphPad Prism 5.0 (GraphPad Software, San Diego, CA). Two-group comparisons were analyzed using unpaired two-tailed *t*-test or non-parametric Mann–Whitney U test for data sets not passing normality or not having equal variances. Experiments with three or more groups were analyzed using one-way ANOVA with the Newman–Keuls multiple comparison test. For all tests, *P* < 0.05 were considered statistically significant (^*^). The number of asterisks indicates the *P* level (^*^ <0.05, ^**^ <0.01, and ^***^ <0.001).

## Results

### Sirt3 Deficiency Is Associated With the Development of TAD

**Figure 1:**
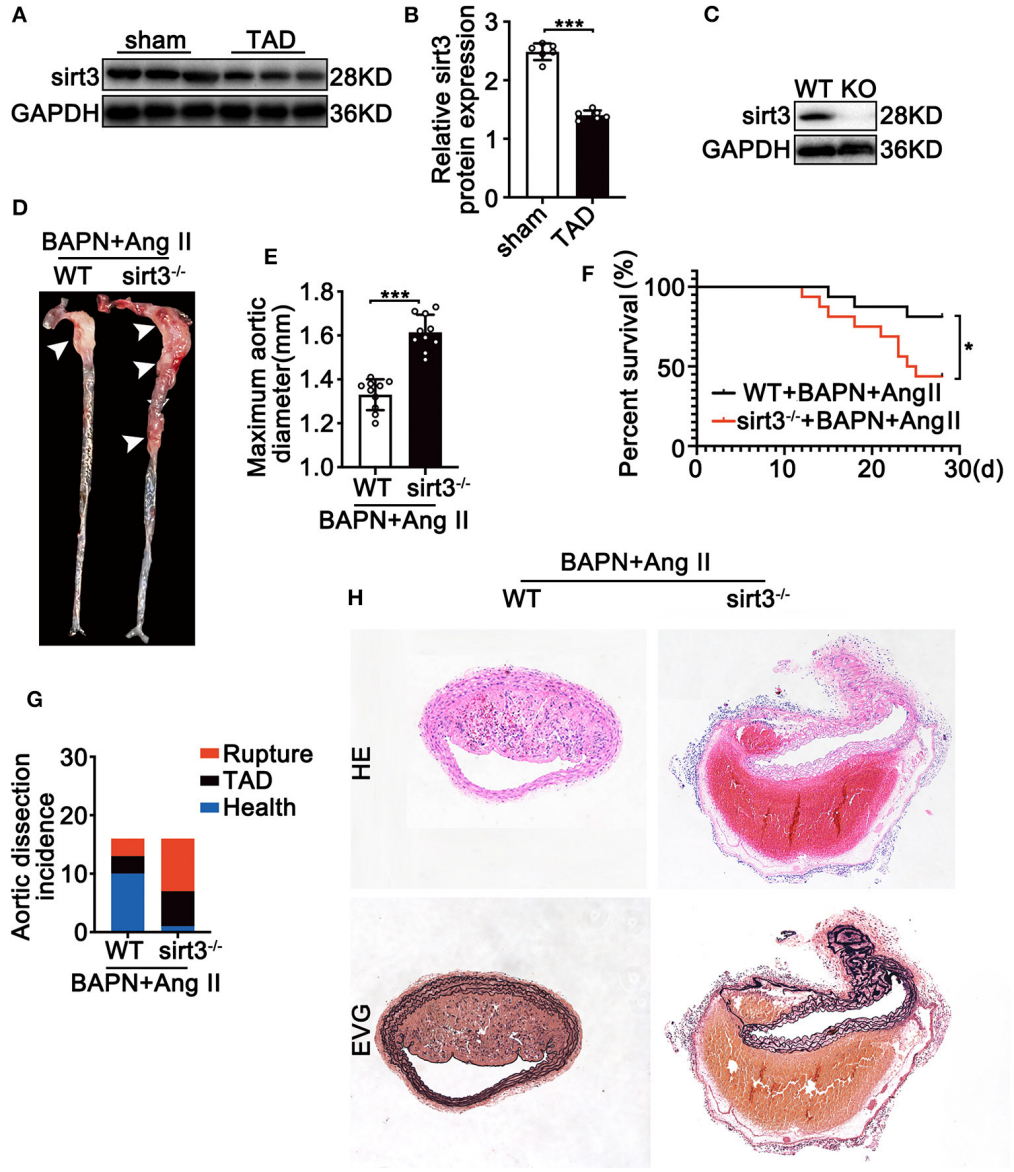
Sirt3 deficiency is associated with the development of TAD in murine model. **(A,B)** Representative Western blot analysis of Sirt3 expression in continuous Ang II (1,000 ng/kg/min) and BAPN (1 g/kg per day) infusion. Data are shown as the mean ± SEM and are representative of six independent experiments, unpaired two-tailed *t*-test, ***P < 0.001. **(C)** Representative Western blot analysis of Sirt3 knockout efficiency using dissecting aortic aneurysm tissues. **(D,E)** Representative figures of the aortas and aorta diameters of WT and Sirt3 KO mice with continuous Ang II and BAPN infusion. Data are shown as the mean ± SEM and are representative of 10 independent experiments, unpaired two-tailed *t*-test, ***P < 0.001. **(F)** Survival curves in indicated groups, *P < 0.05. **(G)** Incidence of Ang II and BAPN-induced TAD (*n* = 16, 16). **(H)** Representative macroscopic images of dissecting aortic aneurysm sections stained with hematoxylin and eosin (HE) and Elastin van Gieson (EVG) from WT and Sirt3 KO mice with continuous Ang II and BAPN infusion. Scale bars: 100 μm.

**Figure 2:**
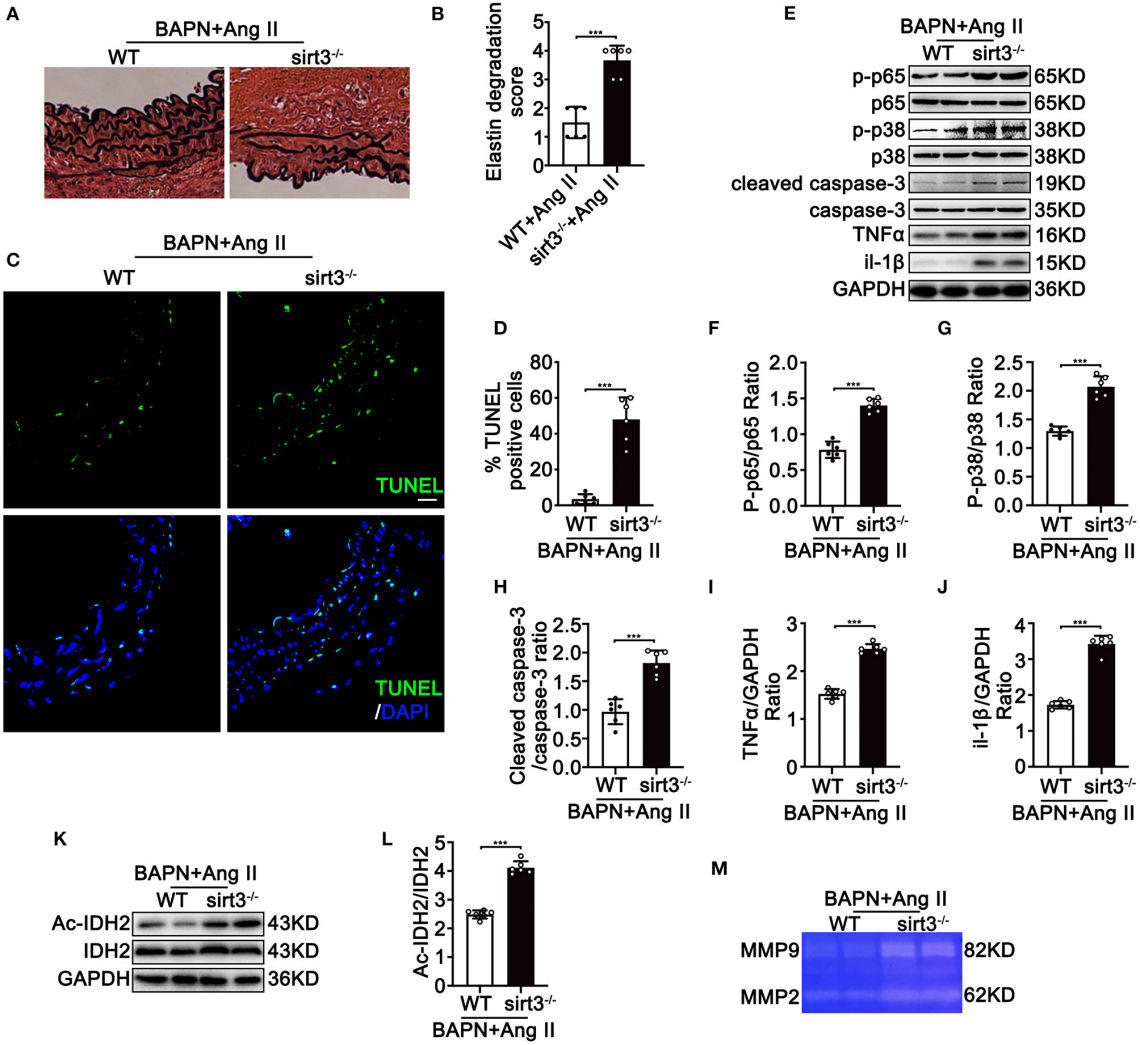
Continuous infusion of Ang II and BAPN in Sirt3 KO mice induces activation of NF-kB, apoptosis, ROS production, and overexpression of MMPs *in vivo*. WT and Sirt3 KO mice with continuous Ang II (1,000 ng/kg/min) and BAPN (1 g/kg per day) infusion for 28 days. **(A,B)** Elastin degradation grading of aortas by EVG staining. *n* = 6. Data are shown as the mean ± SEM and are representative of six independent experiments, unpaired two-tailed *t*-test, ***P < 0.001. (**C,D)** Apoptosis level of aortas by TUNEL assay using fluorescence microscopy. *n* = 6. (**E–J)** Protein expression in the homogenates of aortas by Western blot. *n* = 6. Data are shown as the mean ± SEM and are representative of six independent experiments, unpaired two-tailed *t*-test, ***P < 0.001. **(K,L)** Representative Western blot analysis of acetylated IDH2 and IDH2 in tissues from WT and Sirt3 KO mice with continuous Ang II (1,000 ng/kg/min) and BAPN (1 g/kg per day) infusion. Data are shown as the mean ± SEM and are representative of six independent experiments, unpaired two-tailed *t*-test, ***P < 0.001. **(M)** Representative images of MMP-2 and MMP-9 activity were obtained by a gelatin zymogram using dissecting aortic aneurysm tissue from WT and Sirt3 KO mice that were continuously infused with Ang II and BAPN.

To determine the involvement of Sirt3 in the pathophysiological process of TAD, we first assessed the Sirt3 protein level in ascending aortic tissues from dissecting aortic sites of TAD mice and non-TAD controls. Sirt3 expression (Figures 1A,B) was significantly decreased in ascending aortic tissues from dissecting aortic sites of TAD mice compared with non-TAD controls. To evaluate the roles of Sirt3 in TAD formation, homozygous Sirt3 KO mice (Figure 1C) were subjected to continuous BAPN and Ang II infusion. Sirt3 KO mice have increased aortic diameters and decreased survival rate compared with WT mice (Figures 1D–F). During the 28 days of BAPN infusion, 18.75% WT mice and 56.25% Sirt3 KO mice died of aortic rupture. Meanwhile, 18.75% WT mice and 37.5% Sirt3 KO mice treated with BAPN and Ang II had aortic dissection formation (Figure 1G). HE and EVG staining showed that dissecting aneurysm formation and elastin disarray were also aggravated in Sirt3 KO mice when compared with WT mice treated with BAPN and Ang II (Figure 1H). The elastin degradation score (Figures 2A,B) and SMC apoptosis rates (Figures 2C,D) were increased in sirt3 KO mice compared with those of their littermates. In addition, increased phosphorylation of p65/p65 (Figures 2E,F), phosphorylation of p38/p38 (Figures 2E,G), the levels of cleaved-caspase-3 (Figures 2E,H), and the expression of TNF-α (Figures 2E,I) and IL-1β (Figures 2E,J) were observed in the aortic tissues of Sirt3 KO mice compared with those in WT aortic tissues (Figure 2E). In addition, considering that Sirt3 is a deacetylase, we detected the effect of Sirt3 knockout on IDH acetylation, and the results showed that the degree of IDH acetylation in Sirt3 KO mice was significantly increased compared with WT mice (Figures 2K,L). Gelatin zymogram assay confirmed that in Sirt3-deficient mice the activities of matrix metalloproteinase-2 (MMP-2) and−9 (MMP-9) in aortic dissection tissues were markedly increased (Figure 2M).

### Silencing Sirt3 Leads to Increased Ang II-Induced Phosphorylation of p65, Overexpression of MMP-2 and MMP-9, and Increased ROS Production and Apoptosis in HASMCs *In vitro*

**Figure 3:**
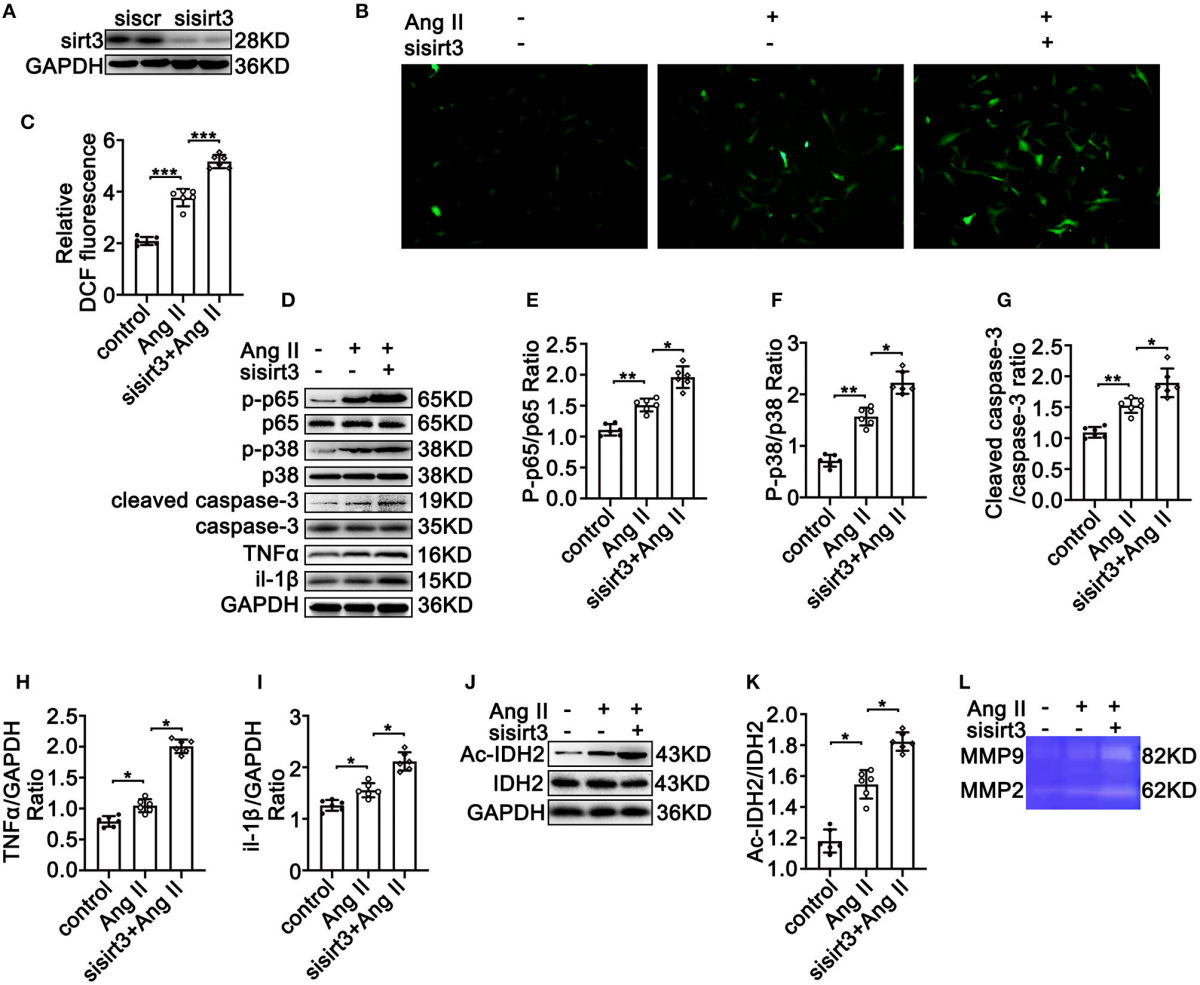
Silencing Sirt3 leads to increased Ang II-induced inflammation-related protein expression, ROS production, and apoptosis in HASMCs *in vitro*. Control siRNA and siSirt3 were transfected into HASMCs with Ang II (100 nM) treatment. **(A)** Western blot analysis verifies the transfection efficiency of sirt3 interfering RNA. **(B,C)** ROS production by DCFH-DA staining and measuring DCF levels using a BioTek Synergy H4 fluorescence microplate reader. *n* = 6. Data are shown as the mean ± SEM and are representative of six independent experiments, one-way ANOVA with Newman–Keuls multiple-comparison test, ***P < 0.001. **(D–K)** Protein expression of phosphorylation of p65, p65, phosphorylation of p38, p38, TNF-α, IL-1β, cleaved-caspase-3, caspase-3, Ac-IDH2, IDH2, and GAPDH by Western blot. Data are shown as the mean ± SEM and are representative of six independent experiments, one-way ANOVA with Newman–Keuls multiple comparison test, *P < 0.05, **P < 0.01. **(L)** Representative images of MMP-2 and MMP-9 activity were obtained by a gelatin zymogram in HASMCs with or without Sirt3 interfere.

To test the crucial roles of Sirt3 in TAD formation, HASMCs were transfected with siSirt3 (Figure 3A) under Ang II conditions following DCFH-DA staining. Silencing Sirt3 increased Ang II induced ROS production (Figures 3B,C). Moreover, silencing Sirt3 promoted Ang II-induced inflammation by increasing the phosphorylation of p65/p65 (Figures 3D,E), and the expression of TNF-α (Figures 3D,H) and Il-1β (Figures 3D,I). Silencing Sirt3 further increased Ang II-induced phosphorylation of p38 (Figures 3D,F) and the level of cleaved-caspase 3 (Figures 3D,G). Compared with the control group, IDH acetylation was significantly increased after silencing Sirt3 (Figures 3J,K). Gelatin zymogram assay showed that the activities of MMP-2 and MMP-9 in HASMCs were remarkably increased after Sirt3 interfere (Figure 3L). Taken together, these results indicated that Sirt3 deficiency led to increased Ang II-induced vascular inflammation, overexpression of MMPs, increased ROS production, and HASMC apoptosis *in vitro*.

### Sirt3 Overexpression Reduces Ang II and BAPN-Induced TAD Formation

**Figure 4:**
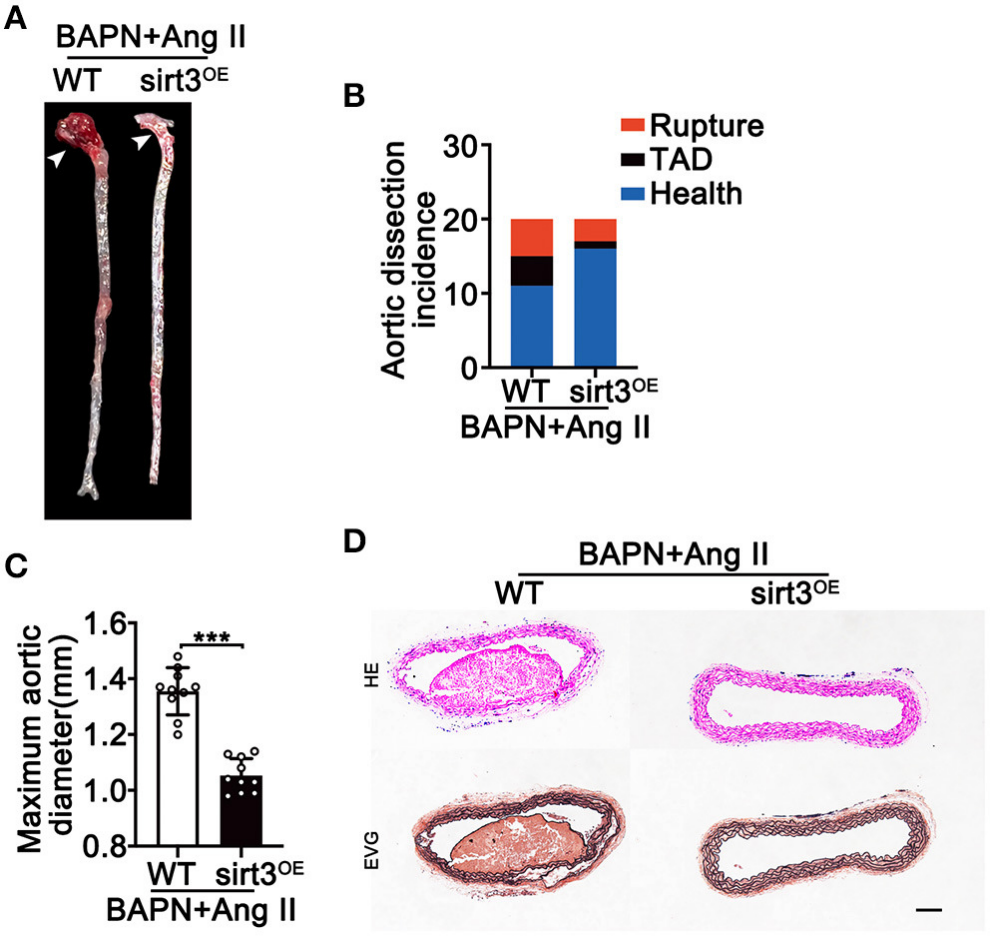
Sirt3 overexpression reduces Ang II and BAPN-induced TAD formation. **(A,C)** Representative figures of the aortas and aorta diameters of WT and Sirt3^OE^ mice with continuous Ang II (1,000 ng/kg/min) and BAPN (1 g/kg per day) infusion for 28 days. Data are shown as the mean ± SEM and are representative of 10 independent experiments, unpaired two-tailed *t*-test, ***P < 0.001. **(B)** Incidence of Ang II and BAPN-induced TAD (*n* = 20, 20). **(D)** Representative macroscopic images of dissecting aortic aneurysm sections stained with HE and EVG from WT and Sirt3^OE^ mice with continuous Ang II and BAPN infusion. Scale bars: 100 μm.

To evaluate the possible effect of Sirt3 overexpression on TAD formation, we induced TAD by continuous Ang II infusion in BAPN treated mice. We found that continuous infusion of Ang II and BAPN induced TAD development in the thoracic aorta; however, overexpression with Sirt3 significantly reduced the incidence of TAD formation (Figures 4A,B). The external aortic diameter was also significantly attenuated by Sirt3 overexpression (Figure 4C). HE and EVG staining showed that dissecting aneurysm formation and elastin disarray were also alleviated in Sirt3 overexpression mice when compared with WT mice treated with BAPN and Ang II (Figure 4D).

### Sirt3 Overexpression Ameliorates Ang II-Induced Phosphorylation of p65, Overexpression of MMP-2 and MMP-9, ROS Production, and Apoptosis in HASMCs *In vitro*

**Figure 5:**
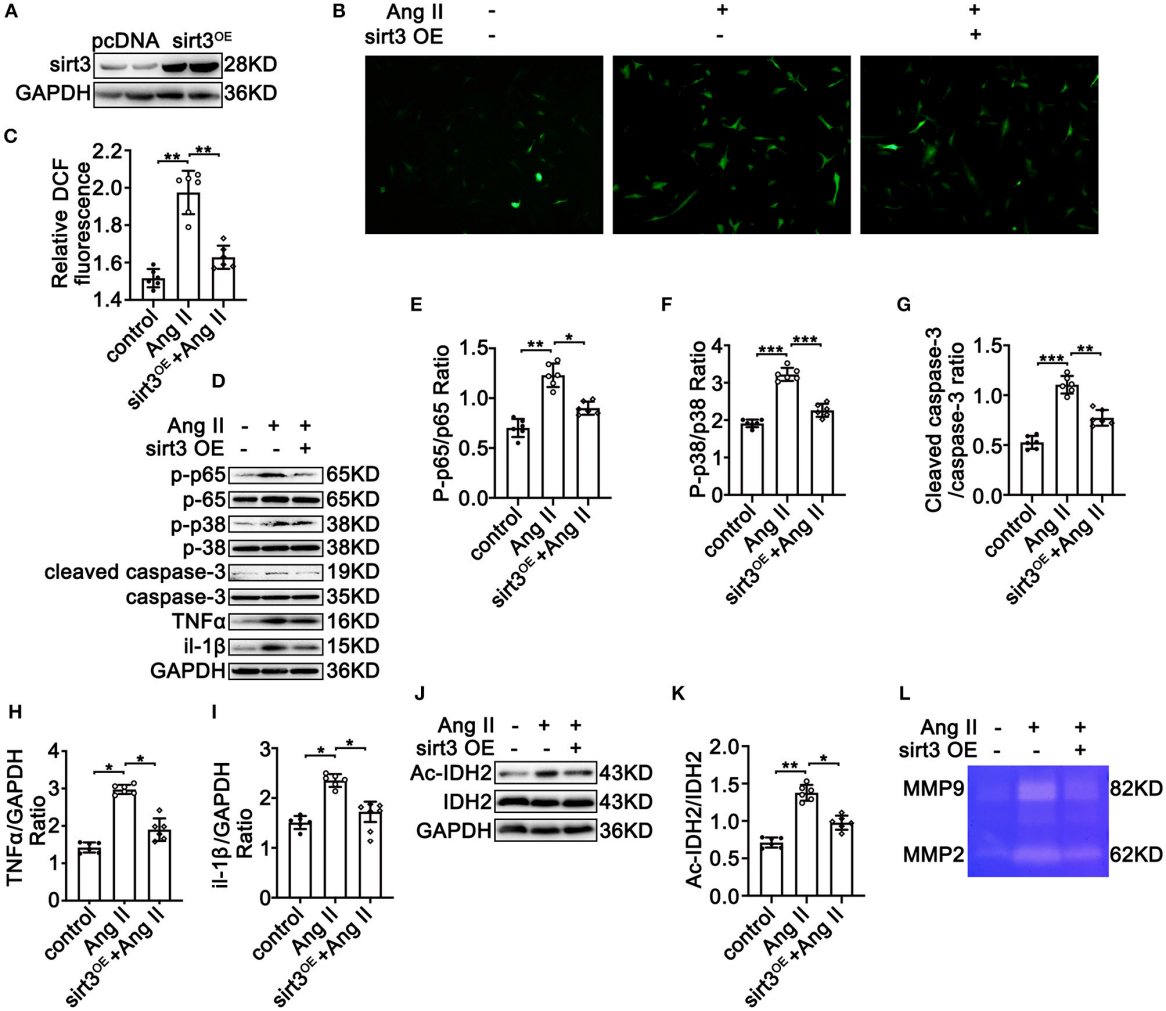
Sirt3 overexpression ameliorates Ang II-induced phosphorylation of p65, overexpression of MMP-2 and MMP-9, ROS production, and apoptosis in HASMCs *in vitro*. pcDNA3.1 and Sirt3 were transfected into HASMCs with Ang II (100 nM) treatment. **(A)** Western blot analysis confirmed the transfection efficiency of the SIRT3-overexpressing plasmid. **(B,C)** ROS production by DCFH-DA staining and measuring DCF levels using a BioTek Synergy H4 fluorescence microplate reader. *n* = 6. Data are shown as the mean ± SEM and are representative of six independent experiments, one-way ANOVA with Newman–Keuls multiple comparison test, ***P < 0.001. **(D–K)** Protein expression of phosphorylation of p65, p65, phosphorylation of p38, p38, MMP-2/9, TNF-α, IL-1β, cleaved-caspase-3, caspase-3, Ac-IDH2, IDH2, and GAPDH by Western blot. Data are shown as the mean ± SEM and are representative of six independent experiments, one-way ANOVA with Newman–Keuls multiple comparison test, *P < 0.05, **P < 0.01. **(L)** Representative images of MMP-2 and MMP-9 activity were obtained by a gelatin zymogram in HASMCs with or without Sirt3 overexpression.

To further decipher the potential protective effects of Sirt3 on TAD formation, HASMCs were overexpressed with Sirt3 (Figure 5A) under Ang II conditions. Overexpressed Sirt3 reduced Ang II-induced ROS production (Figures 5B,C) and inflammation by reducing the phosphorylation of p65/p65 (Figures 5D,E), and the expression of TNF-α (Figures 5D,H) and IL-1β (Figures 5D,I). SMC apoptosis is a cardinal pathological feature of TAD. Overexpressed Sirt3 ameliorated the Ang II and BAPN-induced phosphorylation of p38 (Figures 5D,F) and cleaved-caspase-3 level (Figures 5D,G). IDH acetylation was significantly reduced after Sirt3 overexpression compared with the control groups (Figures 5J,K). The decreased activity of MMP-2 and MMP-9 in HASMCs after Sirt3 overexpression was confirmed by gelatin zymogram (Figure 5L). Taken together, these results indicated that Sirt3 overexpression decreased Ang II-induced vascular inflammation, overexpression of MMPs, ROS production, and HASMC apoptosis *in vitro*.

## Discussion

Recently, animal models of TAD have been established through genetic mutations, surgical procedures, and drug treatment, but the results have been mixed (21). However, long-term use of Ang II can establish a more successful animal model of TAD (22). Therefore, in this study, we also used the Ang II to construct a TAD model. We first found that the expression of Sirt3 was significantly downregulated in the diseased vascular tissues of TAD mice, indicating to a certain extent that Sirt3 is related to TAD. Subsequently, we utilize Sirt3 KO mice constructed by the Chinese Academy of Sciences to clarify the contribution of Sirt3 to the pathogenesis of TAD. The results show that the lack of Sirt3 promotes the development of TAD, including increasing the diameter of dissected blood vessels and increasing the rupture rate of dissected blood vessels. Furthermore, we proved that Sirt3 deficiency mainly promotes the degeneration of the aortic middle layer by enhancing the vascular wall inflammation response and oxidative stress response and promoting SMC apoptosis, which ultimately leads to the occurrence of TAD. In addition, through Sirt3 overexpression experiments, we have proved that Sirt3 overexpression significantly reversed or improved the occurrence and development of TAD. Therefore, these data indicate that the lack of Sirt3 may promote TAD by increasing ROS and SMC apoptosis.

An important pathological change of TAD is the degeneration of the media, and the specific mechanism leading to the degeneration of the media has not been fully elucidated. At present, it is believed that the degradation of ECM in the media layer caused by increased inflammation of blood vessel walls, increased oxidative stress ROS products, and increased MMP activity may be an important mechanism (4). This is very similar to the pathogenesis of aortic aneurysm, another relatively well-studied aortic disease (23). Among them, the research on TAD caused by the increase of ROS caused by mitochondrial function and metabolic disorders has become a hot field in recent years. The main reason is that ROS affects the expression of MMPs and induces SMCs apoptosis, which are also the core pathological changes of TAD and aneurysm (24).

Sirt3 is one of the most important members of the Sirtuin family. It is mainly located in the mitochondria and has strong deacetylase activity. It has a wide range of substrates and is closely related to cardiovascular diseases. In addition, it is also the only member of the Sirtuin family which has been shown to have a life-extending effect in humans (25). We know that normal mitochondrial function is very important for maintaining the physiological processes of the body, and Sirt3 is involved in almost every major aspect of mitochondrial biology, such as fatty acid oxidation, tricarboxylic acid cycle, antioxidant defense, mitochondrial protein synthesis, and mitochondrial dynamics. Sirt3 regulates mitochondrial lysine acetylation and maintains mitochondrial function, participates in mitochondrial biogenesis and stress response, and also detoxifies ROS and reduces oxidative stress damage through various mechanisms (26). Increased mitochondrial ROS due to Sirt3 deficiency is closely associated with many cardiovascular diseases, such as cardiac hypertrophy, myocardial fibrosis, and heart failure (12, 27, 28). However, whether Sirt3 deficiency is involved in the occurrence and development of TAD has not been reported so far.

We utilized Sirt3 KO mice and found that the loss of Sirt3 significantly increased the rupture rate of the dissection. By HE and EVG staining, we observed that the laminar interlayer hematoma in Sirt3-deficient mice was larger in size, with broken elastic fibers, and disorganized in structure compared to normal controls. Previous studies have also shown that mitochondrial disorders mediated by Sirt3 deficiency are involved in the development of human cardiovascular diseases (29). However, through whose mechanism Sirt3 participates in the occurrence and development of TAD, further exploration is needed. By reviewing the studies of Sirt3 and other cardiovascular diseases, we found that in H9C2 cardiomyocytes with Sirt3 overexpression, the opening of the mitochondrial permeability transition pore (MPTP) was restricted due to the deacetylation of CYPD, and the death of cardiomyocytes caused by oxidative damage was reduced (30). In addition, the expression level of Sirt3 mRNA in peripheral blood of patients with heart failure was significantly lower than that of the control group, and it was negatively correlated with blood MDA, 80HdG content, LAD, LVEDD, and LVEDV and positively correlated with SOD content and LVEF, which suggests that when heart failure occurs, the decrease in Sirt3 level may be involved in oxidative stress damage, especially mitochondrial damage and the process of heart remodeling (31). Intervention and treatment of Sirt3 may inhibit oxidative stress damage and delay or even reverse cardiac remodeling in heart failure. Collectively, these studies suggest whether Sirt3 deficiency can also promote the development of TAD through oxidative stress.

As expected, through DCFH-DA staining we found that Sirt3 deficiency can significantly increase the ROS content of smooth muscle cells, while Sirt3 overexpression significantly reduces ROS content, which suggests that oxidative stress does participate in the mechanism of Sirt3 promoting TAD. Oxidative stress can activate the NF-kB pathway, which is a proven important inflammatory response mediator in the pathogenesis of TAD (32). NF-kB enters the nucleus after activation and acts as a transcription factor to enhance the production of inflammatory cytokines, then cytokines further promote the transendothelial migration of circulating monocytes to the middle aorta and differentiate into active macrophages to secrete MMPs and other ECM-degrading proteins to accelerate the formation and progression of TAD. In addition, recent literature has shown that Sirt3 mediates PSG, an anthraquinone extracted from Japanese sorghum, by reducing the expression of NF-kB in activated HSC, and alleviates liver fibrosis through anti-inflammatory effects (33). Koumine is one of the main components of the clover and has been used to treat inflammatory diseases, such as rheumatoid arthritis. The main mechanism is that Koumine can inhibit ROS-NF-kB-related pathways and inhibit the activation of inflammasomes (34). These findings indicate that oxidative stress is closely related to inflammation. Previous literature has shown that Sirt3 has an important protective effect on the pathophysiology of osteoarthritis by inhibiting the PI3K/Akt/mTOR signaling pathway (35). Overexpression of Sirt3 can inhibit IL-1β-induced inflammation, apoptosis, mitochondrial dysfunction, and chondrocyte degeneration; however, knockout of Sirt3 is the opposite (35). Consistent with the previous literature, we observed that the expression of NF-kB was significantly increased in the aortic tissue of Sirt3 knockout mice. In addition, the literature also shows that the lack of Sirt3 can enhance the P38 MAPK pathway. For example, in the experiment of vincristine (VCR) inducing tumor cell apoptosis, one of the mechanisms may be that VCR downregulates the expression of Sirt3, which in turn leads to the accumulation of mitochondrial ROS and initiates the phosphorylation of p38 MAPK and upregulates the expression of TNF-α, which ultimately leads to the apoptosis of U937 and HL-60 cells (36). Moreover, the activation of P38 MAPK also plays an important role in the process of aortic remodeling. Therefore, we also tested the activation of P38 MAPK, and we found that the phosphorylation of P38 MAPK increased after Sirt3 knockout. In addition, the literature shows that the activation of P38 MAPK can upregulate the expression of MMP, maybe by stabilizing the corresponding mRNA transcript, and Ang II treatment can also promote the increase in the expression of MMP2 and MMP9 in vascular smooth muscle cells (37). Similarly, in our experiment, we also found that Sirt3 interference can significantly increase the expression of MMP2 and MMP9 in the aorta, although overexpression of Sirt3 showed the opposite result. Taken together, these results indicate that Sirt3 deletion promotes the pathogenesis of dissection by promoting inflammation and oxidative stress.

Another cause of degeneration of the aortic media is the apoptosis of SMCs (2). Studies have shown that ROS can not only regulate MMPs but also play a key role in inducing SMC apoptosis (24). However, Sirt3 loss can lead to ROS accumulation, and whether it can also cause apoptosis of SMCs is unknown. Through immunofluorescence and WB, we observed that the apoptosis of aortic SMCs in Sirt3 KO mice was significantly increased, and the overexpression of Sirt3 showed the opposite result. This suggests that Sirt3 may reduce SMC apoptosis by reducing the production of mitochondrial ROS.

In summary, we observed that Sirt3 expression was downregulated in the aortic tissues of TAD mice induced by BAPN and Ang-II. In addition; we found that gene deletion or overexpression of Sirt3 can affect the progression of TAD by affecting ROS production, inflammatory response, and SMC apoptosis. At present, TAD, like aortic aneurysm, has a high mortality rate worldwide. It is still urgent clinically to combine the new adjunctive approach within current interventions (1), and our research demonstrated that Sirt3 activation is expected to be a new target and adjuvant method for the treatment of TAD.

## Data Availability Statement

The raw data supporting the conclusions of this article will be made available by the authors, without undue reservation.

## Ethics Statement

The animal study was reviewed and approved by the Ethics Committee of Shandong First Medical University.

## Author Contributions

YH and LQ designed the study and wrote the manuscript. LQ, SY, TY, and YH performed the experiments, provided the materials, performed the measurements, and analyzed the data and critically revised the manuscript. All the authors approved the final version of the manuscript submitted.

## Conflict of Interest

The authors declare that the research was conducted in the absence of any commercial or financial relationships that could be construed as a potential conflict of interest.
